# Changes in the Demographic and Clinicopathological Characteristics of Thyroid Cancer: A Population-Based Investigation in Algeria, 1993-2013

**DOI:** 10.1155/2020/7812791

**Published:** 2020-09-22

**Authors:** Houda Boukheris, Arslan Bettayeb, Lesley Ann Anderson, Zineb Achour, Fatma Zohra Benbachir, Sarra Attar, Hafida Saim, Kada Rouigeb, Necib Berber

**Affiliations:** ^1^Department of Epidemiology and Preventive Medicine, University Hospital of Bejaia, Algeria; ^2^University of Bejaia, School of Medicine, Algeria; ^3^Department of Epidemiology and Preventive Medicine, University Hospital of Oran, Algeria; ^4^Centre for Public Health and Northern Ireland Cancer Registry, Queen's University Belfast, Northern Ireland, UK; ^5^Aberdeen Centre for Health Data Science, University of Aberdeen, Scotland, UK; ^6^Department of Epidemiology and Preventive Medicine, University Hospital of Tlemcen, Algeria; ^7^Department of Nuclear Medicine, University Hospital of Tlemcen, Algeria

## Abstract

Over the last three decades, the incidence of thyroid cancer has increased worldwide. The reasons for this increase remain controversial. In Algeria, however, to date, information on thyroid cancer has been limited to a hospital-based case series. We analyzed data from a population-based cohort study in Oran District, Algeria, to describe demographic and clinicopathological characteristics of patients diagnosed with thyroid cancer between 1993 and 2013. Medical records and pathology reports of thyroid cancer patients who had surgery were reviewed. Changes in demographic and clinicopathological features over the 21-year period are described. During the study period, thyroid cancer was diagnosed in 1248 women (86.5%, mean age 43.7 ± 15.2 years) and 195 men (23.4%, mean age 48.1 ± 15.9 years). Most cases (83.1% for women and 69.8% for men) sought a diagnosis following a self-neck check. The most common histologic types were papillary (58.3%), follicular (29.7%), anaplastic (4.1%), and medullary (0.8%) carcinomas. The incidence of papillary carcinomas significantly increased (*p* < 0.001) while the incidence of other histologic types significantly decreased over time. Tumor size overall significantly decreased (*p* < 0.001) while the frequency of small (≤20 mm) and larger (>20 mm) carcinomas significantly increased (*p* < 0.05). The frequency of thyroid cancers with capsular effractions and angioinvasions also decreased over time. Thyroid cancer incidence in Algeria has increased substantially in line with international trends with changes in clinical practice being a possible contributing factor. However, the increasing papillary-to-follicular cancer ratio may be due to changes in iodine nutrition status in Algeria. Further research, including exploration of biological and molecular features of thyroid cancer, will enable a better understanding of risk factors and etiopathogenetic mechanisms.

## 1. Background

Thyroid cancer (TC), although rare, is the most common cancer affecting the endocrine system [1, 2]. In 2018, 567233 new TC cases were reported worldwide [3]. Incidence rates are substantially higher among women (10.2 per 100000) than among men (3.1 per 100000) [3]. More than 95% of TCs are derived from follicular cells being classified as differentiated, poorly differentiated (PDTC), and anaplastic (ATC) TCs. The vast majority of differentiated TCs are papillary (PTC) (70% to 90% of TC) or follicular (FTC) (5%-10% of TC) carcinomas [2]. PTC and FTC have a good prognosis with 10-year survival rates of 80%-95% and 70%-90%, respectively, whereas PDTC and ATC, which represent the other 5%-10% of TCs, have much poorer prognosis [2, 4].

In Africa, PTC and FTC are the predominant histologic types with a high proportion of FTCs (52.7% and 35.0%, respectively) [5, 6]. This is not unexpected as in areas with iodine deficiency and endemic goiter, such as Africa, FTC is common [5]. Over the last three decades, TC incidence has sharply increased in many parts of the world, particularly the incidence of PTC and carcinomas ≤ 20 mm, while the incidence of other histologic subtypes has either declined or remained stable [7–21]. The reasons for this are still debated. Some authors have suggested that the increased TC incidence is due to an increased use of thyroid ultrasound detecting small tumors, while others suggest that changes in etiological factors may play a role [21–23]. Among all risk factors identified, exposure to ionizing radiation, especially in childhood, is the only clearly established and well-quantified risk factor for TC, particularly PTC [24–26]. Other potential risk factors include iodine deficiency and endemic goiter [27–30], genetic susceptibility [31, 32], hormonal and reproductive factors [33–35], obesity [36–38], and most recently exposure to endocrine disruptors [39].

According to the International Agency for Research on Cancer, in 2018, TC was the third most common cancer among women and the eighth among men in Algeria [3]. TC incidence was 8.1 per 100000 in women and 1.9 per 100000 in men, with regional variations (6.2 per 100000 to 9.1 per 100000 for women and 1.4 per 100000 to 2.1 per 100000 for men). Over the last three decades, a steady increase in TC incidence has been observed in the region of Setif in the east of Algeria, known for its iodine deficiency and goiter endemicity [40]. However, the clinical and histologic profile of TC in Algeria has never been described, and most epidemiologic studies have been based on a clinical series. We therefore used population-based data on TC, collected in the district of Oran, Northwestern Algeria, to provide information on TC incidence with a focus on its demographic, clinical, and histological features.

## 2. Population and Methods

### 2.1. Study Design

We retrospectively reviewed the medical records and pathology reports of TC patients who underwent thyroid surgery between 1993 and 2013 in healthcare institutions in the district of Oran and analyzed patterns and trends of demographic and clinicopathological characteristics over the 21-year period.

### 2.2. Case Definition

TC diagnosis is anatomopathological based on microscopic examination of the thyroid specimen. Morphological subtypes were defined according to definitions of the World Health Organization (WHO): all TC cases (topographic code C73.9) [41] were grouped into 6 major morphological subtypes according to the International Classification of Diseases for Oncology, third edition (ICD-O-3) [42]. PTC (ICD-O-3 codes: 8050, 8260, 8341-8344, 8350, and 8450-8460), FTC (ICD-O-3 codes: 8190, 8290, and 8330-8335), papillary carcinoma of follicular variant (PTCFV) (ICD-O-3 code: 8340), PDTC (ICD-O-3 code: 8300), ATC (ICD-O-3 codes: 8012 and 8020-8035), MTC (ICD-O-3 codes: 8345, 8346, 8347, and 8510-8513), carcinomas with no other specification (carcinoma NOS) (ICD-O-3 codes: 8000, 8010-8015, 8230, and 8337), and other specified carcinomas (ICD-O-3 codes: 8052, 8333, 8337, 8070, 8140, 9591, and 8800). FVPTC cases were grouped with PTC, and PDTC with ATC. The incidence date was determined using the definition developed by the European Network of Cancer Registries [43].

### 2.3. Data Collection and Validation

As no dedicated clinicopathological database for TC exists in Oran, we collected data using a multisource approach to enhance data completeness and validity as recommended by the ENCR for population-based cancer registries [44]. Information on clinically diagnosed TC patients was abstracted from medical records and pathology reports of patients who underwent thyroid surgery between 1 January 1993 to 31 December 2013, from 43 healthcare institutions across Oran, including public and private clinics and pathology laboratories. To enhance data completeness, we extended our search to all healthcare institutions involved in the diagnosis, management, treatment, and follow-up of TC, nationwide. Our active search focused on TC patients diagnosed with benign thyroid conditions, including thyroid cold nodules and goiters and Grave's disease, and patients with clinically or cytologically suspicious lesions who underwent either total or near-total thyroidectomy followed by microscopic examination of the thyroid specimen. We performed multiple crossovers between the different data sources to validate the histopathological diagnosis and the place of residence of each patient. Only patients with histologically confirmed TC and who were permanent residents of the district of Oran at the time of their TC diagnosis were included in the cohort. As pathologists do not report the place of residence on pathology reports, TC cases that were identified through pathology reports only were crossed with electoral lists in the municipalities to confirm residency. Information of interest included age at TC diagnosis (or date of birth), sex, personal history of benign diseases or malignancies, circumstances of TC diagnosis, date and type of initial thyroid surgery (total or near-total thyroidectomy), the presence of thyroid nodules (TN) and their number, tumor size, capsular effractions, angioinvasions, histologic type, and thyroid benign condition associated with TC. All data were compiled in the same Excel file, and duplicates excluded. For all TC patients included in the study, a pathologist conducted a central review of pathology reports in order to reascertain histological diagnosis.

### 2.4. Statistical Analysis

#### 2.4.1. Definition of Variables

Variables considered in the analysis included age at diagnosis (years), sex, circumstances of TC diagnosis, personal history of thyroid diseases, incidence date, period of TC diagnosis (1993-1997, 1998-2002, 2003-2007, and 2008-2013), major histologic subtypes (6 groups: PTC, FTC, ATC, MTC, other carcinomas specified, and carcinoma NOS), the presence of thyroid nodules and/or cysts (yes/no), their number (solitary (1) versus multiple nodules (2+)), and tumor size at its largest diameter in millimeters. When multifocality was detected, the size of the largest tumor was taken into account in the analysis. Tumor size was analyzed as a continuous variable and then subclassified into categories (first in 4 categories (≤10 mm, 11-2 mm, 21-40 mm, and >40 mm) and then in 2 categories (≤10 mm and >10 mm)), and TC localization (on the nodule or in the parenchyma), thyroid parenchyma invasion (yes/no), effraction of the thyroid capsule (yes/no), and the presence of vascular emboli (yes/no) were observed. Age at TC diagnosis and tumor size were presented as means with the standard deviation (SD) and medians with extreme values, respectively. Data were presented for the entire study period and for the 4 calendar time periods. Categorical and continuous data were tested on univariate analysis using two-tailed Fisher's exact test, independent *t*-tests between groups of two, and one-way analysis of variance (ANOVA) among groups of three or more. Dichotomous outcomes were analyzed using the chi-square test. After checking for the normality assumption, the mean and SD were used to express normally distributed data (such as age of the patients) with a median and interquartile range used for nonnormally distributed data (such as tumor size). A *p* value < 0.05 was considered to indicate statistical significance.

Annual populations for years 1993 to 2013 for the district of Oran (5-year age groups) were obtained from the National Office of Statistics, Algeria (http://www.ons.dz/IMG/pdf/CH1-DEMOGRAPHIE.pdf). Age-standardized incidence rates (ASRs) of TC were computed by the direct standardization method using the world standard population as a reference [42] and were expressed per 100000 person-years. ASRs were calculated for the entire cohort and by histology to assess temporal trends. The data were managed using Microsoft Excel 2010 and analyzed using SPSS for Windows version 20.0 (SPSS Inc., Chicago, IL). ASRs were computed using SEER∗Stat version 6.4.4 [45].

## 3. Results

Between 1993 and 2013, 17324 thyroid surgeries were performed in Oran across the 43 healthcare institutions, among which 7521 were permanent residents of Oran. Major data sources were surgery services (*n* = 17), pathology laboratories (*n* = 11), medical oncology clinics (*n* = 2), radiotherapy clinics (*n* = 2), endocrinology clinic (*n* = 1), and the nuclear medicine facility (*n* = 1).

**Figure 1:**
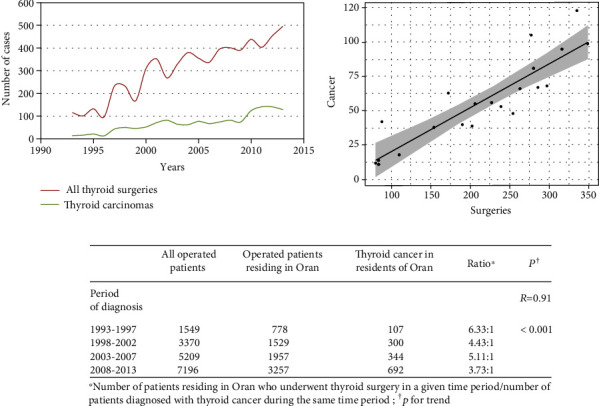
Trends of thyroid surgeries performed among residents of Oran, 1993-2013.

**Figure 2:**
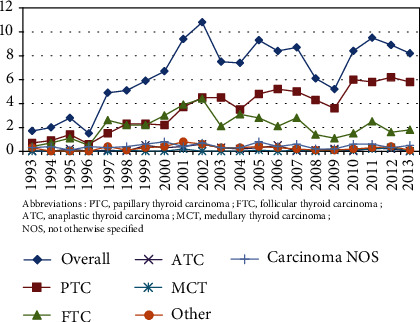
Age-standardized (world population) thyroid cancer incidence, overall and for the major histologic types. Abbreviations: PTC: papillary thyroid carcinoma; FTC: follicular thyroid carcinoma; ATC: anaplastic thyroid carcinoma; MCT: medullary thyroid carcinoma; NOS: not otherwise specified.

After validation of place of residence, histologic diagnosis, and elimination of duplicates, 1443 TC cases were diagnosed over the study period (19.2% of all thyroid surgeries). TC data sources were represented by the nuclear medicine facility (52.5%), pathology laboratories (27.0%), and surgery services (16.6%). An increased number of thyroid surgeries were observed over the study period, concomitant with an increase in the number of thyroid cancer cases detected (Figure 1). The ratio of thyroid surgeries to TC narrowed significantly with time, which reflects a more rapid increase in the frequency of TC compared with the number of thyroid surgeries (1 TC out of 3.73 thyroid surgeries in 2008-2013 versus 1 TC out of 6.33 thyroid surgeries in 1993-1997, *p* < 0.001). Thyroid cancer age-standardized incidence rates also showed a trend towards increased incidence of overall TC and of PTC, while FTC decreased and other histologic types remained relatively stable (Figure 2). Among all TC patients, 1248 (86.5%) were women and 195 (13.5%) men (female-to-male (F : M) sex ratio = 6.4 : 1), with no significant variations over time (*p* = 0.76). Mean age at TC diagnosis (±SD) was 43.7 ± 14.8 years for women and 48.1 ± 15.3 years for men (*p* < 0.001).

A self-neck check was the most common circumstance of diagnosis (83.1% for women and 69.8% for men) with a significant increase in frequency over the study period although for men, it was not statistically significant (71.9% in 1993-1997 to 86.8% in 2008-2013 (*p* = 0.01) for women and 33.3% in 1993-1997 to 78.3% in 2008-2013 for men (*p* = 0.11)) (Table 1). A history of goiter or nodules was found in 10% of women and 18.9% of men, decreasing nonsignificantly over time (15.8% in 1993-1997 to 7.5% in 2008-2013 for women (*p* = 0.07) and 33.3% in 1993-1997 to 18.1% in 2008-2013 for men (*p* = 0.09)) (Table 1). An incidental diagnosis, following medical imaging of the neck, represented 1.2% of cases in women and 10.1% in men, with a significant decrease over time for men (33.3% in 1993-1997 to 3.6% in 2008-2013, *p* < 0.01), while for women, there was no clear trend (Table 1).

In 84.5% of women and 87.8% of men, there was no reported history of thyroid diseases, with significant decreases over time (Table 1). The proportion of TC patients who reported a history of goiter significantly increased over time (13.3% in 1993-1997 to 23.3% in 2008-2013 (*p* < 0.001) for women and 2.9% in 1993-1997 to 25.0% in 2008-2013 (*p* < 0.01) for men). Based on 24 cases only, a significant trend towards an increased number of patients with thyroiditis was observed among females (3.3% in 1993-1997 to 5.0% in 2008-2013, *p* < 0.01) (Table 1).

For women and men, microscopic examination of the thyroid specimen led to the diagnosis of thyroid cold nodules (TCN) (49.7% and 47.1%, respectively), goiter (43.2% and 47.7%, respectively), and thyroiditis (6.5% and 5.1%, respectively), with no significant variations over time (Table 1).

### 3.1. Anatomopathological Characteristics

Thyroid nodules (TNs) were present in 93.5% of women and 90.3% of men (Table 2). The frequency of TCs associated with TN increased over time, although for men, the difference was not statistically significant (81.1% in 1993-1997 to 95.1% in 2008-2013 (*p* < 0.001) for women and 80.8% in 1993-1997 to 87.7% in 2008-2013 (*p* = 0.2) for men). This increase was driven by an increase in the frequency of thyroid multiple nodules, significant only for women (21.7% in 1993-1997 to 40.7% in 2008-2013 (*p* < 0.01) and 20.0% in 1993-1997 to 34.0% in 2008-2013 (*p* = 0.81)) (Table 2).

The mean size of the TCs was 26.4 ± 17.3 mm in women and 29.7 ± 19.1 mm in men (*p* = 0.14). For women and men, microcarcinomas represented 21.3% and 16.5%, respectively, and small carcinomas (≤20 mm) represented 51% and 46.9% of TCs, respectively, while carcinomas > 40 mm represented 14.0% and 21.5%, respectively. Significant differences in TC distributions according to TC size and period of diagnosis were observed. TCs > 20 mm were predominant in 1993-2002, and TCs ≤ 20 mm in 2008-2013 for women (*p* < 0.001). For men, similar distributions were observed for TC ≤ 20 mm, while a higher frequency of TC > 20 mm was observed in 1998-2013 (*p* = 0.03). Over the study period, TC mean size significantly decreased for women (32.6 ± 19.4 mm in 1993-1997 to 24.4 ± 16.8 mm in 2008-2013) (*p* < 0.01), while for men, TC size significantly increased (*p* = 0.048) (Table 2). The frequency of microcarcinomas significantly increased for women (6.1% in 1993-1997 to 25.2% in 2008-2013, *p* < 0.001), while a nonsignificant decrease in the frequency of TC > 40 mm was observed (21.2% in 1993-1997 to 11.6% in 2008-2013, *p* = 0.15) (Table 2).

Cancerous nodules accounted for 58.5% of TCs in women and 46.6% in men, sparing the thyroid parenchyma in 82.6% of women and 77.6% of men (Table 2). For women, the frequency of TCs sparing the thyroid parenchyma significantly increased over time (41.7% in 1993-1997 to 87.9% in 2008-2013, *p* < 0.001), while for men, a significant decrease was observed during the same time period (50% in 1998-2002 to 8% in 2008-2013, *p* = 0.025) (Table 2). Capsular effractions were present in 57.7% of women and 54.2% of men, and angioinvasions in 50.1% of women and 46.6% of men with a significant decrease in frequency over time (Table 2).

For women and men, TC histologic types were represented by PTC (59.5% and 51.8%), FTC (30.4% and 25.6%), carcinoma NOS (5.1% and 9.7%), ATC (1.6% and 5.1%), and MCT (0.8% and 1.0%) (Table 2). Over the study period, the frequency of PTCs significantly increased (from 42.7% to 69.8% in women (*p* < 0.001) and 23.1% to 67.0% in men (*p* < 0.001)), while a significant decrease was observed for FTC, carcinoma NOS, ATC, and all other specified carcinomas. PTCFV accounted for 16.3% and 6.3% of all histologic types in women and men, respectively, with no significant variations across the study period (Table 2).

## 4. Discussion

To our knowledge, this is the first study to use population-based data to describe demographic, clinical, and pathological patterns and trends of TC in an African country. From 1993 to 2013, 1443 new TC cases were diagnosed among permanent residents in the district of Oran, Algeria, of which 1248 (86.5%) were women (sex ratio: 6.4 : 1). The self-neck check was the most frequent circumstance of diagnosis and increased over time. No prior history of thyroid disease was reported in more than 85% of patients. TNs were observed in more than 90% of TC patients, and their frequency increased over the study period. The most frequent TC histologic types were PTC, FTC, and carcinoma NOS. Over the study period, the frequency of PTC increased while that of FTC, ATC, and carcinoma NOS decreased. Tumor size significantly decreased, with an increased frequency of microcarcinomas.

Consistent with data from African cancer registries, TC occurred in our cohort during the fourth decade of life [5]. However, in recent years, some studies have reported an increased incidence of TC among younger individuals in developed countries [8, 14]. TC rates were higher in women compared with men in Oran (F : M ratio 6.4 : 1). A F : M ratio of 5.9 : 1 was also reported in a previous study carried out in two different districts in eastern Algeria (Guidoum et al.). Worldwide, the F : M ratio for TC incidence varies from 2 to 12 [3], and this disparity by sex has been attributed to hormonal factors and greater use of healthcare services by women compared with men [11, 33–35].

The trend towards increased incidence of TC observed in our study has also been reported in two different regions covered by population-based cancer registries with cancer data collected over two decades [40, 46]. Socioeconomic differences relating to access to healthcare services have been hypothesized to influence TC incidence and trends [17]. However, this should not influence TC rates or trends in Oran as free access to healthcare and universal insurance coverage (public funded with no dependency on income level) were implemented in Algeria in 1974. Thus, the general population can be considered equal regarding the amount and quality of healthcare available, suggesting that a detection effect would not fully explain the observed increase in TC cases. Furthermore, since the early 1990s, the private sector has expanded dramatically and private clinics have mushroomed. Our finding that 96% of TC patients in the cohort have undergone thyroid surgery in public hospitals contrasts with the higher frequency of microscopic examination of thyroid specimens performed in private pathology laboratories (92%), which may reflect easy access to healthcare services in both the public and private services. Oran is the second largest and one of the most developed cities in Algeria, a context that may have favored the increasing number of individuals seeking healthcare across the study period and undergoing thyroid surgery. Higher levels of education, greater awareness of the disease in the general population, and the increased frequency of thyroid surgery over the study period may also explain part of the observed changes over time. Three major changes in medical practice have occurred in Oran over the study period and may explain part of the observed trends: (1) large thyroidectomies were performed more frequently over the past two decades and may have led to the incidental discovery of small TC, (2) thyroid ultrasound was introduced in 1990, (3) and fine-needle aspiration was introduced in 1998. Furthermore, all medical procedures were standardized across the clinics making TC patients equal regarding TC diagnosis and management.

The overall increase in TC in Oran was mainly due to a rise in the frequency of PTC, while the frequency of other histologic types decreased. A diagnostic effect alone would have resulted in similar increases for all histologic types. Other factors that vary with time may have contributed to the observed trends, for example, iodine intake [30], obesity [36–38], exposure to endocrine disruptors [39], use of fertility drugs [47], hormonal and reproductive factors [33–35], and insulin resistance syndrome [48, 49]. In Algeria, apart from iodine intake, obesity, and diabetes, little is known about the prevalence of the other potential risk factors.

Iodine deficiency and supplementation may increase the risk of benign thyroid diseases, which may lead to increased rates of thyroid surgery and incidental discovery of subclinical TC [50]. In addition, differences in risk according to the histologic type have also been associated with iodine intake [20, 21]. When iodine supplementation is introduced in a population with a previous background of iodine deficiency, there is a shift towards an increase in the PTC-to-FTC ratio within 15 to 40 years, with no actual change in the overall TC incidence [30]. Algeria was iodine-deficient with a high prevalence of endemic goiter [50]. In 1967, the program of table salt iodization was implemented, first in areas with high prevalence of endemic goiter and nationwide since 1990 [51]. In our cohort, distributions of PTC and FTC were similar in 1993-1997. However, over the study period, the frequency of PTC increased while that of FTC decreased, which may reflect a shift from iodine-deficient to iodine sufficient-to-excessive supplementation status. In a previous publication by Guidoum et al., PTC was the predominant histologic type in two districts in Northeastern Algeria [52].

In our study, a decrease in frequency of tumor aggressiveness features (capsular effractions and angioinvasions) over time is consistent with a shift from FTC to the less aggressive PTC and also early diagnosis in the context of iodine supplementation.

The prevalence of TC in TN varies from 4% to 6.5% [30] and is less frequent in multinodular goiters than in solitary nodules among residents of regions with iodine deficiency [30]. In our study, the prevalence of TC was substantially higher at 19.2%. Among TC patients, 90.9% exhibited either goiter or TCN. Goiters and nodules are considered precancerous lesions that share risk factors with TC such as iodine deficiency and exposure to ionizing radiation [2]. The significant increase in the prevalence of TN and chronic thyroiditis across the study period may be associated with an excessive nutritional iodine intake as reported in studies conducted in other countries [30]. The increased number of thyroid surgeries over the study period, with a systematic use of total and near-total thyroidectomy in the context of high prevalence of goiter and benign TNs, may have helped detect microcarcinomas in the studied population. In our study, mean size of TC decreased among females but increased in males. A few studies found a significant increase in small TC over time in both women and men, but TC of larger sizes also increased among men [7, 11, 23]. In France between 1983 and 2000, incidence of PTC 10-40 mm increased from 0.38 per 100000 to 0.83 per 100000 in men and from 0.05 per 100000 to 0.17 per 100000 for PTCs > 40 mm [11]. In Italy, significant increases in small TCs were observed in 2001-2006; however, TCs > 20 mm also increased (APC: +18.4%) [23]. In the U.S. for the period 1980-2005, 20% of the TC increase was due to tumors > 20 mm, and in 1988-2009, TC incidence of TC > 20 mm significantly increased [7]. While in developed countries, increased TC incidence over the past several years has been attributed to the use of medical imaging of the neck resulting in an epidemic of incidentalomas [20–23], incidental detection of TC in the course of medical imaging in our study occurred in 1.2% of women and 10.1% of men and decreased over time, while the frequency of incidental discovery during microscopic examination of the thyroid specimen by pathologists increased.

Obesity increases the risk of TC [36–38]. Insulin resistance and subsequent hyperinsulinemia are present in 50% of patients with PTC [48, 49], and a high BMI has increased the risk of TC in women [36–38]. The rapid increase in the prevalence of obesity and diabetes has paralleled the increase in incidence of TC, in particular PTC in women [38]. In our cohort, among the 298 TC patients with anthropometric data, 64 (21.3%) had a BMI > 33 kg/m^2^ and 12 (4%) were overweight. Changes in eating habits and lifestyle have resulted in an increased prevalence of obesity in Algeria. In 2010, the prevalence of obesity among adults aged 35 to 70 years was 30.1% (95% CI: 27.8%-32.4%) in women and 9.1% (95% CI: 7.1%-11.0%) in men [53]. The prevalence of diabetes among adults in Oran has increased between 1998 and 2009, from 7.1% to 10.5% in Oran [54].

Strengths of this study include the large number of TC cases evaluated in an unselected and homogeneous population. Oran is the second largest and most populated city in Algeria (about 1.5 million inhabitants in 2014—about 5% of the Algerian population). We have used the ENCR definitions and the multisource approach to reach a high degree of completeness of case ascertainment across the studied period. Only TC cases with microscopic confirmation and validated place of residence were included in the study. Data collection processes also included a systematic recovery of demographic, clinical, and anatomopathological features and tumor size. In 1988, the WHO new classification of morphologies considered the PTCFV to be PTC. Since our study included patients diagnosed from 1993 onwards, we do not expect that this revision has influenced the observed trends for PTC. Also, the proportions of PTCFV were similar in the 4 time periods assessed. The accuracy and completeness of information on histology were also high resulting in lower frequencies of cases with poorly specified TC histology in later years, which reflects improved classification of poorly specified histology by pathologists over time. Despite our efforts to ensure data quality and completeness, a few data limitations should be acknowledged. We failed to capture TC patients treated in medical centers outside of the district of Oran, but these patients represented less than 5% of the cohort. In our cohort, we failed to find medical records and pathology reports of all TC patients diagnosed during the early period of the study, and information on clinical characteristics, tumor size, nodules, and other microscopic characteristics was not complete. The significant decrease in missing data over time may reflect better data capture and could explain part of the increasing trends concerning clinical characteristics and some pathological features. However, it is unlikely that these limitations have invalidated our study, because the observed trends are consistent with the general context that includes a history of iodine deficiency and endemic goiter and the high frequency of nodules and goiter in previously asymptomatic patients, and our findings concerning trends of histologic types, tumor size, and demographics align well with previously published data.

In summary, our study revealed a sharp increase in TC incidence that has tripled over the study period, and this trend has paralleled an increased frequency of thyroid surgery, particularly in women. The increased number of patients seeking healthcare spontaneously is compatible with the hypothesis of the change in consumer behaviour and greater awareness of thyroid pathology in the general public, probably due to improvements in education. Striking observations include a rise in TC cases limited to PTC, with decreasing proportions of other histologic types, in particular FTC. The increasing PTC-to-FTC ratio over the study period suggests that differences in iodine nutrition status over time might have affected the observed trends. In conclusion, it is not possible to identify the exact cause of the increasing TC trends in Algeria. Increased diagnostic activity may have also played a role. Future large-scale research will need to include information on TC patients' anthropometric characteristics, tumor size, and TC stage to determine the role of iodine prophylaxis among other factors in PTC and FTC patterns and trends.

## Figures and Tables

**Table 1:** Demographic and clinical characteristics of thyroid cancer patients according to sex and the period of diagnosis.

Characteristics	Total (*n* = 1 248)	1993-1997 (*n* = 96)	1998-2002 (*n* = 255)	2003-2007 (*n* = 297)	2008-2013 (*n* = 600)	*p* ^∗^	Total (*n* = 195)	1993-1997 (*n* = 13)	1998-2002 (*n* = 44)	2003-2007 (*n* = 47)	2008-2013 (*n* = 91)	*p* ^∗^
	Women						Men					
Sex ratio (F : M)	6.4 : 1	7.4 : 1	5.8 : 1	6.3 : 1	6.6 : 1	0.76						
Mean age (yrs) (±SD)^∗∗^	43.7 ± 14.8	38.9 ± 15.9	43.8 ± 14.3	44.4 ± 15.2	44.2 ± 15.3	0.48^†^	48.1 ± 15.3	47.3 ± 11.9	47.4 ± 17.2	49.8 ± 15.2	47.5 ± 14.9	0.37
Median (Min–Max)	43 (11-94)	36 (13-80)	43 (14-79)	43 (15-94)	42 (12-85)		49 (11-81)	51 (20-60)	49 (11-81)	49 (24-80)	46 (28-79)	
Circumstances of diagnosis^‡^	(*n* = 827)	(*n* = 57)	(*n* = 141)	(*n* = 205)	(*n* = 424)	0.08^†^	(*n* = 169)	(*n* = 9)	(*n* = 34)	(*n* = 43)	(*n* = 83)	0.049^†^
Self-neck check	687 (83.1)	41 (71.9)	113 (80.1)	165 (80.5)	368 (86.8)	0.01	118 (69.8)	3 (33.3)	22 (64.7)	29 (67.4)	65 (78.3)	0.11
Goiter/nodules	83 (10.0)	9 (15.8)	16 (11.3)	26 (12.7)	32 (7.5)	0.07	32 (18.9)	3 (33.3)	6 (17.6)	9 (20.9)	15 (18.1)	0.9
Medical exam	47 (5.7)	6 (10.5)	11 (7.8)	13 (6.3)	17 (4.0)	0.09	2 (1.2)	0 (0)	0 (0)	0 (0)	0 (0)	NA
Medical imaging	10 (1.2)	1 (1.8)	1 (0.7)	1 (0.5)	7 (1.7)	0.57	17 (10.1)	3 (33.3)	6 (17.6)	5 (11.6)	3 (3.6)	<0.01
Unknown^#^	421 (33.7)	39 (40.6)	114 (44.7)	92 (30.9)	176 (29.3)		26 (13.3)	4 (30.8)	10 (22.7)	4 (8.5)	8 (8.8)	
History of thyroid diseases^§^	(*n* = 761)	(*n* = 30)	(*n* = 176)	(*n* = 233)	(*n* = 322)	<0.001^†^	(*n* = 123)	(*n* = 6)	(*n* = 35)	(*n* = 38)	(*n* = 44)	<0.01^†^
None	643 (84.5)	25 (83.3)	168 (95.5)	219 (94.0)	231 (71.7)	<0.001	108 (87.8)	6 (100)	34 (97.1)	36 (94.7)	32 (72.7)	<0.01
Goiter^¶^	94 (12.4)	4 (13.3)	6 (3.4)	9 (3.9)	75 (23.3)	<0.001	14 (11.4)	0 (0)	1 (2.9)	2 (5.2)	11 (25.0)	<0.01
Thyroiditis^§^	24 (3.2)	1 (3.3)	2 (1.1)	5 (2.1)	16 (5.0)	<0.001	1 (0.8)	0 (0)	0 (0)	0 (0)	1 (2.3)	NA
Unknown^#^	487 (39.0)	66 (68.7)	79 (30.9)	64 (21.5)	278 (46.3)		72 (36.9)	7 (53.8)	9 (20.4)	9 (19.1)	47 (51.6)	
Pathological diagnosis^ǁ^	(*n* = 1125)	(*n* = 90)	(*n* = 233)	(*n* = 271)	(*n* = 531)		(*n* = 174)	(*n* = 11)	(*n* = 42)	(*n* = 45)	(*n* = 76)	
TCN	559 (49.7)	44 (48.9)	119 (51.1)	124 (45.8)	272 (51.2)	0.07^†^	82 (47.1)	3 (27.3)	24 (57.1)	11 (24.4)	44 (57.9)	<0.01^†^
Goiter^¶^	486 (43.2)	39 (43.3)	96 (41.2)	124 (45.8)	227 (42.7)	0.75	83 (47.7)	8 (72.7)	17 (40.5)	28 (62.2)	30 (39.5)	0.32
Thyroiditis^§^	80 (6.5)	7 (7.8)	18 (6.9)	23 (8.5)	32 (5.1)	0.82	9 (5.1)	0 (0)	1 (2.4)	6 (13.3)	2 (2.6)	0.65
Unknown^#^	123 (9.8)	6 (2.3)	22 (8.6)	26 (8.7)	69 (11.5)	0.15	21 (10.8)	2 (15.4)	2 (4.5)	2 (4.2)	15 (16.5)	0.60

Abbreviations: *n*: frequencies; F: females; M: males; yrs: years; SD: standard deviation; Min: minimum; Max: maximum; TCN: thyroid cold nodules. ^∗^*p* statistical test for trend; *p* value showing difference between groups excluding unknown (^∗∗^Student's *t*-test). Men were significantly older than women (*p* < 0.001). ^†^Variance analysis. ^‡^*p* for heterogeneity (females versus males). ^§^*p* for heterogeneity (females versus males). ¶ included colloid goiters, parenchymatous goiters, multinodular goiters, and multinodular heterogengoiters. ǁ includes Grave's disease, Hashimoto thyroiditis, lymphocytic thyroiditis, and de Quervain's thyroiditis.

**Table 2:** Pathologic characteristics of thyroid cancer patients according to sex and the period of diagnosis.

Characteristics	Total (*n* = 1 248)	1993-1997 (*n* = 96)	1998-2002 (*n* = 255)	2003-2007 (*n* = 297)	2008-2013 (*n* = 600)	*p* ^∗^	Total (*n* = 195)	1993-1997 (*n* = 13)	1998-2002 (*n* = 44)	2003-2007 (*n* = 47)	2008-2013 (*n* = 91)	*p* ^∗^
	Women						Men					
Nodules	(*n* = 980)	(*n* = 74)	(*n* = 167)	(*n* = 252)	(*n* = 487)		(*n* = 144)	(*n* = 10)	(*n* = 28)	(*n* = 41)	(*n* = 65)	
Yes	916 (93.5)	60 (81.1)	153 (91.6)	240 (95.2)	463 (95.1)	<0.001	130 (90.3)	8 (80.0)	27 (96.4)	38 (92.7)	57 (87.7)	
No	64 (6.5)	14 (18.9)	14 (8.4)	12 (4.8)	24 (4.9)		14 (9.7)	2 (20.0)	1 (3.6)	3 (7.3)	8 (12.3)	0.2
Unknown	268 (21.5)	22 (22.9)	88 (34.5)	45 (15.1)	113 (18.8)	NS	51 (26.1)	3 (23.1)	16 (57.1)	6 (12.8)	26 (28.6)	NS
Number of nodules	(*n* = 741)	(*n* = 46)	(*n* = 104)	(*n* = 203)	(*n* = 388)		(*n* = 90)	(*n* = 5)	(*n* = 10)	(*n* = 28)	(*n* = 47)	
1	463 (62.5)	36 (78.3)	75 (72.1)	122 (60.1)	230 (59.3)	<0.01	62 (68.9)	4 (80.0)	8 (80.0)	19 (67.9)	31 (66.0)	
≥2	278 (37.5)	10 (21.7)	29 (27.9)	81 (39.9)	158 (40.7)		28 (31.1)	1 (20.0)	2 (20.0)	9 (32.1)	16 (34.0)	0.81
Unknown	507 (40.6)	36 (37.5)	137 (53.7)	82 (27.6)	188 (31.3)	<0.05	105 (53.8)	8 (61.5)	33 (77.3)	19 (40.4)	44 (48.3)	NS
Tumor size (mm)						<0.01^†^						0.048^†^
Mean ± SD	26.4 ± 17.3	32.6 ± 19.4	30.1 ± 17.7	27.4 ± 17.2	24.4 ± 16.8		29.7 ± 19.1	8.5^†^ ± 2.1	27.1 ± 18.1	36 ± 13.2	28.2 ± 19.4	
Median (Min–Max)	20 (2-100)	30.6 (3-80)	30.0 (5-85)	23.5 (2-90)	20 (2-100)		25 (3-70)	(7-10)	21 (10-65)	31 (12-70)	20 (3-70)	
	(*n* = 649)	(*n* = 33)	(*n* = 83)	(*n* = 172)	(*n* = 361)	<0.001^†^	(*n* = 79)	(*n* = 3)	(*n* = 10)	(*n* = 22)	(*n* = 44)	0.03^†^
≤10	138 (21.3)	2 (6.1)	13 (15.7)	32 (18.6)	91 (25.2)	0.014	13 (16.5)	2 (66.7)	3 (30.0)	0 (0)	8 (18.2)	<0.01
11-20	193 (29.7)	11 (33.3)	15 (18.1)	50 (29.1)	117 (32.4)	0.072	24 (30.4)	0 (0)	2 (20.0)	7 (31.8)	15 (34.1)	0.74
21-40	227 (35.0)	13 (39.4)	39 (47.0)	64 (37.2)	111 (30.7)	0.036	25 (31.6)	1 (33.3)	3 (30.0)	8 (36.4)	13 (29.5)	0.81
>40	91 (14.0)	7 (21.2)	16 (19.3)	26 (15.1)	42 (11.6)	0.15	17 (21.5)	0 (0)	2 (20.0)	7 (31.8)	8 (18.2)	0.56
Unknown	599 (47.9)	8 (8.3)	158 (61.9)	113 (38.0)	215 (35.8)	<0.05	102 (52.3)	8 (61.5)	33 (75.0)	22 (46.8)	39 (42.8)	NS
Carcinoma location	(*n* = 738)	(*n* = 46)	(*n* = 128)	(*n* = 175)	(*n* = 389)		(*n* = 161)	(*n* = 8)	(*n* = 31)	(*n* = 28)	(*n* = 51)	
Nodule	432 (58.5)	25 (54.3)	67 (52.3)	107 (61.1)	233 (59.9)	0.18	75 (46.6)	4 (50.0)	19 (61.3)	21 (75.0)	31 (60.8)	
Parenchyma	306 (41.5)	21 (45.7)	61 (47.7)	68 (38.9)	156 (40.1)		43 (26.7)	4 (50.0)	12 (38.7)	7 (25.0)	20 (39.2)	0.45
Unknown	446 (35.7)	36 (37.5)	113 (44.3)	110 (37.0)	187 (31.2)	<0.05	63 (32.3)	3 (23.1)	12 (27.3)	16 (34.0)	32 (35.1)	NS
Parenchyma status	(*n* = 288)	(*n* = 12)	(*n* = 33)	(*n* = 69)	(*n* = 174)		(*n* = 49)	(*n* = 2)	(*n* = 10)	(*n* = 12)	(*n* = 25)	
Not invaded	238 (82.6)	5 (41.7)	22 (66.7)	58 (84.1)	153 (87.9)	<0.001	38 (77.6)	0 (0)	5 (50.0)	4 (33.3)	2 (8.0)	
Invaded	50 (17.4)	7 (58.3)	11 (33.3)	11 (15.9)	21 (12.1)		11 (22.4)	2 (100)	5 (50.0)	8 (66.7)	23 (92.0)	0.025
Unknown	590 (47.3)	49 (51.0)	147 (57.6)	148 (49.8)	246 (41.0)	<0.05	89 (45.6)	5 (38.5)	21 (47.7)	25 (53.2)	38 (41.7)	NS
Capsular effractions	(*n* = 631)	(*n* = 49)	(*n* = 92)	(*n* = 156)	(*n* = 334)	<0.001	(*n* = 83)	(*n* = 8)	(*n* = 6)	(*n* = 23)	(*n* = 46)	NS
Yes	364 (57.7)	27 (55.1)	65 (70.7)	118 (75.6)	154 (46.1)	<0.001	45 (54.2)	7 (87.5)	5 (83.3)	15 (65.2)	18 (39.1)	0.012
No	267 (42.3)	22 (44.9)	27 (29.3)	38 (24.4)	180 (53.9)		38 (45.8)	1 (12.5)	1 (12.5)	8 (34.8)	28 (60.9)	NS
Unknown	617 (49.4)	47 (48.9)	163 (63.9)	141 (47.5)	266 (44.3)	<0.05	112 (57.4)	7 (53.8)	36 (81.8)	24 (51.1)	45 (49.5)	NS
Angioinvasions	(*n* = 563)	(*n* = 42)	(*n* = 80)	(*n* = 150)	(*n* = 2911)		(*n* = 73)	(*n* = 5)	(*n* = 8)	(*n* = 20)	(*n* = 40)	
Yes	282 (50.1)	21 (50.0)	49 (61.2)	107 (71.3)	105 (36.1)	<0.001	34 (46.6)	4 (80.0)	7 (87.5)	10 (50.0)	13 (32.5)	
No	281 (49.9)	21 (50.0)	31 (38.8)	43 (28.7)	186 (63.9)		39 (53.4)	1 (20.0)	1 (12.5)	10 (50.0)	27 (67.5)	<0.01
Unknown	685 (54.9)	54 (56.2)	175 (68.6)	147 (49.5)	309 (51.5)	<0.05	122 (62.5)	8 (61.5)	36 (81.8)	27 (57.4)	51 (56.0)	NS
Histological subtype	(*n* = 1248)	(*n* = 96)	(*n* = 255)	(*n* = 297)	(*n* = 600)		(*n* = 195)	(*n* = 13)	(*n* = 44)	(*n* = 47)	(*n* = 91)	
Papillary^‡^	742 (59.5)	41 (42.7)	107 (42.0)	175 (58.9)	419 (69.8)	<0.001	101 (51.8)	3 (23.1)	12 (27.3)	25 (53.2)	61 (67.0)	<0.001
CPFV	204 (16.3)	17 (17.7)	35 (13.7)	53 (17.8)	99 (16.5)	0.24	13 (6.7)	0 (0)	2 (4.5)	3 (6.4)	8 (8.8)	0.33
Follicular	379 (30.4)	41 (42.7)	115 (45.1)	93 (31.3)	130 (21.7)	<0.001	50 (25.6)	6 (46.1)	16 (36.3)	10 (21.3)	18 (19.8)	0.046
Carcinoma NOS	64 (5.1)	9 (9.4)	14 (5.5)	14 (4.7)	27 (4.5)	0.26	19 (9.7)	3 (23.1)	6 (13.6)	5 (10.6)	5 (5.5)	0.096
Anaplastic^§^	20 (1.6)	2 (2.1)	7 (2.7)	4 (1.3)	7 (1.2)	0.016	10 (5.1)	0 (0)	5 (11.4)	4 (8.5)	1 (1.1)	0.027
Medullary	10 (0.8)	0 (0)	2 (0.8)	2 (0.7)	6 (1.0)	NA	2 (1.0)	0 (0)	0 (0)	0 (0)	2 (2.2)	NA
Others^II^	33 (2.6)	3 (3.1)	10 (3.9)	9 (3.0)	11 (1.8)	<0.001	13 (6.7)	1 (7.7)	5 (11.4)	3 (6.4)	4 (4.4)	<0.001

Abbreviations: *n*: frequencies; mm: millimeters; SD: standard deviation; Min: minimum; Max: maximum. ^∗^*p* statistical test for trend. *p* value showing difference between groups excluding unknown (^∗∗^Student's *t*-test). Men were significantly older than women (*p* < 0.001). ^†^Variance analysis. ^‡^*p* for heterogeneity (women versus men). ^§^*p* for heterogeneity (women versus men). ¶ included colloid goiters, diffuse goiters, multinodular goiters, and multinodular heterogengoiters. ǁ includes Graves' disease, Hashimoto thyroiditis, lymphocytic thyroiditis, and de Quervain's thyroiditis.

## Data Availability

The work described in the submitted article is the first part of my PhD dissertation that I have not defended yet. Thus, I cannot share my data immediately with the general public or the scientific community. I am open to share all the data related to this work after my PhD dissertation has taken place.

## Additional Points

*What Is New?*. This is a cohort study of 1443 Algerian patients diagnosed with thyroid cancer over a 21-year period. Most patients sought a diagnosis following a self-neck check. Thyroid nodules were associated with thyroid cancer in 90% of patients. The incidence of the papillary carcinoma histotype significantly increased over time, while that of the follicular histotype significantly decreased. Tumor size significantly decreased over time, but the frequency of larger tumors increased. The frequency of thyroid cancers with capsular effractions and angioinvasions decreased over time. The increasing papillary-to-follicular carcinoma ratio may be due to changes in iodine nutrition status in Algeria, although changes in medical practice cannot be ruled out.
